# Diagnostic and Prognostic Values of miRNAs in High-Grade Gliomas: A Systematic Review

**DOI:** 10.12688/f1000research.151350.2

**Published:** 2025-01-15

**Authors:** Renindra Ananda Aman, Mohammad Galih Pratama, Ricky Rusydi Satriawan, Irfani Ryan Ardiansyah, I Ketut Agus Suanjaya

**Affiliations:** 1 Department of Neurosurgery, Faculty of Medicine, University of Indonesia, Jakarta, DKI Jakarta, Indonesia

**Keywords:** miRNA, Glioma, Biomarker, Prognostic, Oncogene

## Abstract

**Background:**

Gliomas, particularly glioblastomas, have grim prognoses, necessitating early diagnostic and prognostic indicators. MicroRNAs (miRNAs), influential in cancer research, show potential as glioma biomarkers. This systematic review aimed to examine the efficacy of miRNAs in the diagnosis and prognosis of high-grade glioma.

**Methods:**

A comprehensive search was conducted of PubMed, EMBASE, Cochrane Library, and Web of Science for studies published from 2013 to 2023. The eligibility criteria included high-grade glioma, histopathological confirmation, miRNA samples from cerebrospinal fluid or plasma, and relevant outcome data. Studies were excluded if they were experimental or reviews and not in English.

**Results:**

Of the 1120 initial results, 8 studies involving 660 subjects met the inclusion criteria. Several studies have assessed miRNA expression and its association with diagnosis and prognosis of high-grade gliomas. Overexpression of miR-221, miR-222, miR-210, miR-21, miR-125b, and miR-223 and under-expression of miR-15b and miR-124-3p showed significant potential in differentiating high-grade glioma patients from controls. Additionally, miRNAs are associated with distinct tumorigenic pathways.

**Conclusion:**

Elevated or depressed expression levels of specific circulating miRNAs hold significant promise as noninvasive biomarkers for the diagnosis and prognosis of high-grade glioma. These miRNAs offer valuable insights into disease progression and patient outcome. Further validation through extensive clinical trials and in-depth mechanistic studies is essential to realize their full clinical utility.

## 1. Introduction

Gliomas, a class of tumors originating in the central nervous system, pose a formidable medical challenge given their grim prognosis and complex nature. Glioblastoma is the most prevalent form among adults, with an incidence rate of 6.34 cases per 100,000 individuals. ^1^ This aggressive tumor is characterized by rapid growth, often progressing for weeks or months. Despite the implementation of a multimodal therapeutic approach involving surgery, radiotherapy, and chemotherapy, the average survival for glioblastoma patients is only 15 months. ^2^ Against this backdrop of clinical urgency, the search for early diagnostic and prognostic indicators for gliomas is of paramount importance. Gliomas are the most common and aggressive type of primary brain tumor in adults. It should be noted that, for gliomas, malignancy (e.g., anaplastic astrocytoma and glioblastoma) can arise de novo or progress from the lower grade. Despite all the achievements of modern medicine, the prognosis for patients with high-grade gliomas remains unsatisfactory. ^3^

MicroRNAs (miRNAs) have emerged as a captivating subject of study within the realm of cancer research, owing to their intricate involvement in the disease’s intricate molecular landscape. Comprising a class of small, non-coding RNAs composed of 19 to 22 nucleotides, miRNAs have a powerful influence on gene expression and cellular processes. ^4^ Of note is their role in tumorigenesis, where alterations in miRNA target binding sites and disruptions in the miRNA processing machinery within tumor cells have been identified as significant contributors to cancer development and progression. ^5^

This burgeoning field of research has delved into the depth of miRNA expression patterns in gliomas, yielding captivating insights into their diagnostic and prognostic potential. A growing body of evidence underscores the intricate correlations between specific miRNAs and the clinical aspects of gliomas, which have shown promise not only as potential biomarkers for early detection but also as indicators of disease progression and therapeutic response. ^5^^–^^8^ By elucidating the complex web of miRNA-glioma associations, researchers aim to unlock a treasure trove of information that could ultimately revolutionize the diagnosis, treatment, and management of this relentless disease. This review aims to examine the effectiveness of miRNAs in the diagnosis and prognosis of high-grade gliomas.

## 2. Methods

### 2.1 Search strategy

We carefully searched the literature using online free accessed software Rayyan ( https://new.rayyan.ai/) with literature databases such as PubMed ( https://pubmed.ncbi.nlm.nih.gov/), EMBASE ( https://www.embase.com/landing?status=grey), Cochrane Library ( https://www.cochranelibrary.com/), and Web of Science ( https://www.webofscience.com/wos) to identify relevant studies published between 2013 and 2023 using the following strategy:

(((((microrna) OR (mirna)) OR (mirnas) AND (y_5[Filter])) AND (((((high grade glioma) OR (glioblastoma)) OR (anaplastic astrocytoma)) OR (anaplastic oligodendroglioma)) OR (anaplastic oligoastrocytoma) AND (y_5[Filter]))) AND (((diagnostic) OR (diagnosis)) OR (early diagnosis) AND (y_5[Filter]))) AND ((prognostic) OR (survival) AND (y_5[Filter])) Filters: in the last 5 years.

### 2.2 Eligibility criteria

The selection process for studies to be included in this study adhered to stringent eligibility criteria. Studies were deemed eligible if they satisfied the following criteria: high-grade gliomas, including glioblastoma, anaplastic astrocytoma, anaplastic oligodendroglioma, and anaplastic oligoastrocytoma, as confirmed by histopathological examination. miRNA samples were collected from cerebrospinal fluid or plasma. For prognostic studies, the inclusion criterion required survival curves depicting overall survival (OS), disease-free survival (DFS), cause-specific survival (CSS), or recurrence-free survival (RFS). These survival curves were expected to be accompanied by hazard ratios (HR) and the corresponding 95% confidence intervals (CIs). Conversely, diagnostic studies are required to present data encompassing true positive, true negative, false positive, and false negative outcomes.

Studies were excluded if they were categorized as experimental studies, review articles, or letters. Studies that were not conducted on human subjects and those that were not published in English were excluded. These rigorous inclusion and exclusion criteria were used to ensure the selection of studies that met the stringent standards of scientific rigor and relevance for research. Flow chart showing study selection process will be displayed at result section.

### 2.3 Data extraction and quality assessment

Two independent authors collected the study characteristics and original data, including the first author’s name, publication year, study design, study population, population size, age and sex of participants, follow-up duration, sample type, method of measuring miRNA expression, and HRs and their 95% CIs. Studies were included according to the following checklist based on the criteria provided by the MOOSE group: clearly defined study design; clearly described study population (country); sufficiently large sample (N>30); clearly described outcome (OS, CSS, DFS, or RFS); clearly defined miR measurement, including quantitative real-time polymerase chain reaction (qRT-PCR) or in situ hybridization (ISH); clear definition of cut-off values; miR measurement in tumor tissue, plasma, or serum; and sufficiently long follow-up. Studies that did not meet these criteria were excluded. Quality assessment was performed using the Cohcrane’s risk of bias in non-randomized studies of interventions (ROBINS-I) tool. Any discrepancies regarding the risk of bias judgments were resolved by discussion until agreement was reached.

Certainty of evidence refers to the confidence that the observed effects of mRNA expression levels and their implications in glioma accurately reflect their true biological and clinical impact. Using the GRADE framework, factors such as study design, risk of bias, consistency of results, directness of evidence, precision of estimates, and publication bias were assessed meticulously. A high certainty of evidence suggests that further research is unlikely to significantly alter the current understanding, whereas low certainty indicates that new studies could substantially impact the conclusions. This rigorous evaluation ensured that the systematic review provided a reliable foundation for advancing research and informing clinical practices related to mRNA and glioma.

## 3. Results

### 3.1 Overview of study selection

**Figure 1.**
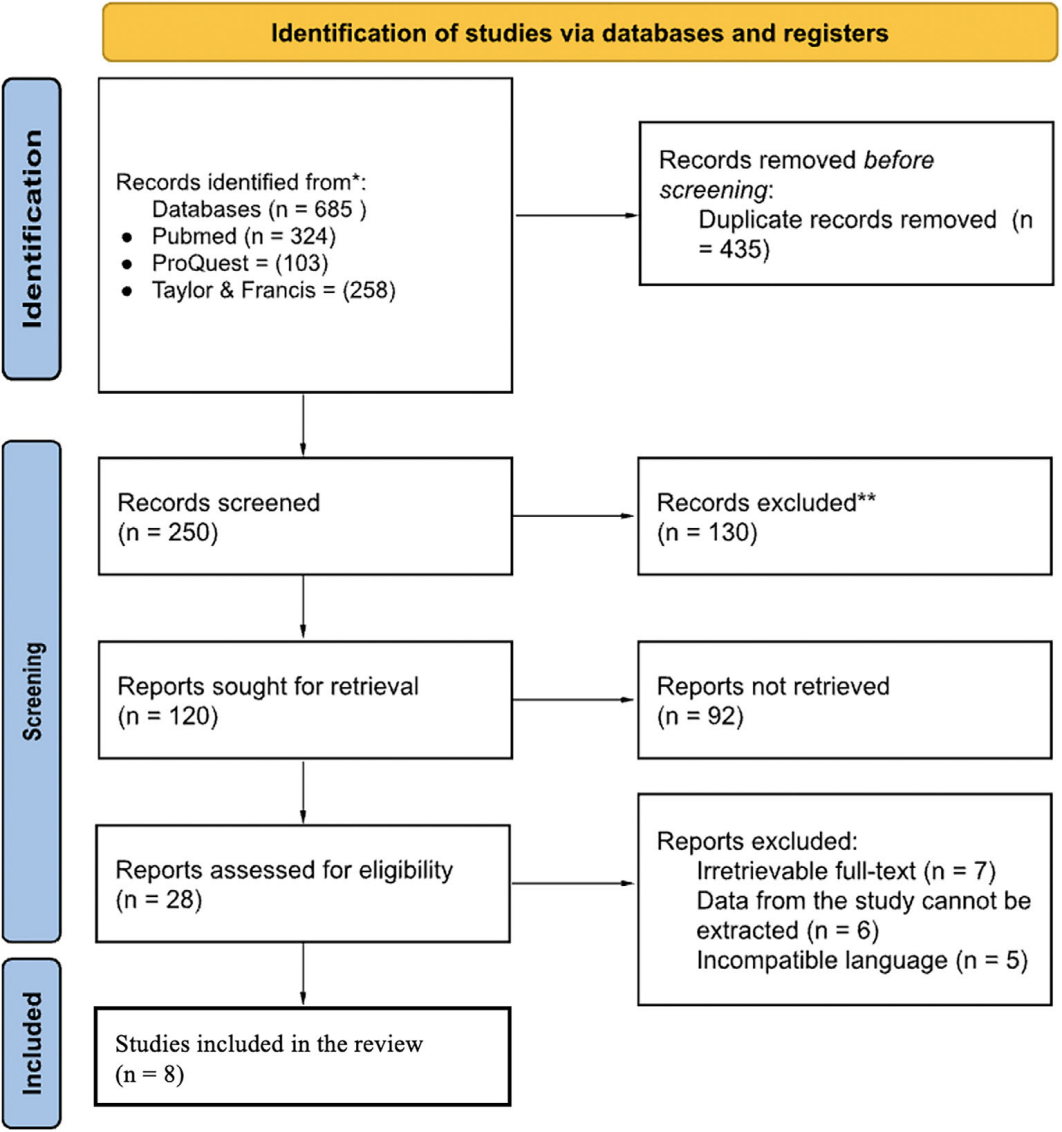
PRISMA study selection process diagram.

A Literature search yielded 1120 results, of which 8 studies met the criteria, with a total of 660 subjects. The complete search process is illustrated in Figure 1.

### 3.2 Characteristics of included studies

**Figure 2.**
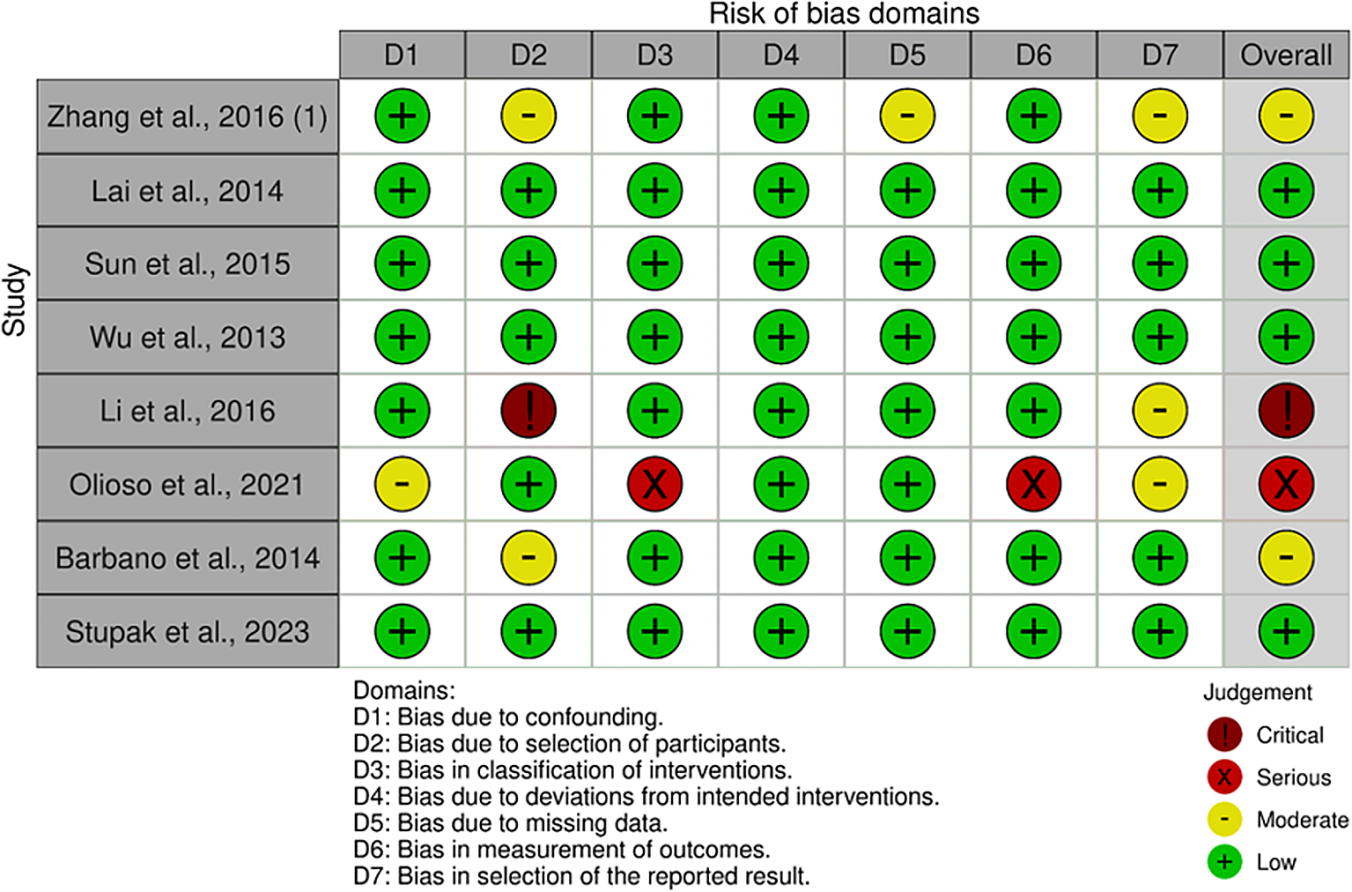
Results of study quality assessment using the ROBINS-I tool (a) domain specific quality assessment graph.

The quality assessment of each study is shown in Figure 2. The characteristics of the included studies are presented in Table 1. A total of 660 participants were included in this study. The sample size of each study ranged from 39 to 152 patients. The majority of the studies (n = 5) were conducted in China, and the remainder were conducted in, Italy (n = 2), and Russia (n = 1). One study conducted observations and follow-ups after 48, 60, > 60, and > 100 weeks. Most studies have used qRT-PCR to detect and measure the expression levels of miRNAs. In addition to diagnosis, the study assessed prognosis by calculating HRs and 95% Cis. A summary of each miRNA, its potential targets, and pathways is presented in Table 2.

**Table 1.** Characteristics of Studies in Included in Our Review.

miRNA	Study	Country	n Sample	Sample source	Control (n)	Measurement	WHO Classification	Follow-up	Hazard ratio	95% CI	p-value
miR-221	Zhang et al, 2016 ^9^	China	50	Peripheral venous blood	Venous blood from non-glioma patient (50)	qRT-PCR	2007	48	2.4	1.42-4.05	0.001
miR-222	Zhang et al, 2016 ^9^	China	50	Peripheral venous blood	Venous blood from non-glioma patient (50)	qRT-PCR	2007	48	2.81	1.70-4.65	0.001
miR-210	Lai et al, 2014 ^10^	China	125	Glioma tissue	Brain tissue from non-glioma patient ^10^	qRT-PCR	2007	>100	2.3	1.47-3.61	0.003
miR-15b	Sun et al, 2015 ^11^	China	92	Glioma tissue	Brain tissue from non-glioma patient ^12^	qRT-PCR	2007	>60	2.21	1.36-3.6	0.001
miR-21	Wu et al, 2013 ^12^	China	152	Glioma tissue	Adjacent non-glioma tissue (152)	qRT-PCR	2007	60	3.17	2.39-4.18	0.001
miR-125b	Li et al, 2016 ^13^	China	45	Glioma tissue	Adjacent non-glioma tissue (45)	qRT-PCR	2007	>100	2.43	1.13-2.54	0.023
miR-21	Olioso et al, 2021 ^14^	Italy	57	Peripheral venous blood	Peripheral venous blood of the patient at follow up (57)	qRT-PCR	2007	>60	0.4	0.13-1.43	0.017
miR-222	Olioso et al, 2021 ^14^	Italy	57	Peripheral venous blood	Peripheral venous blood of the patient at follow up (57)	qRT-PCR	2007	>60	0.4	0.13-1.38	0.003
miR-124-3p	Olioso et al, 2021 ^14^	Italy	57	Peripheral venous blood	Peripheral venous blood of the patient at follow up (57)	qRT-PCR	2007	>60	0.4	0.13-1.38	0.022
miR-21	Barbano et al, 2014 ^15^	Italy	32	Glioma tissue	Brain tissue from non-glioma patient ^4^	qRT-PCR	2007	>60	1.19	1.01-1.41	0.04
miR-223	Stupak et al, 2023 ^16^	Russia	107	Glioma tissue	Adjacent non-glioma tissue (107)	qRT-PCR	2007	48	1.1	1.00-1.22	0.046

**Table 2.** Summary of miRNA, potential targets, and pathways.

miRNA	Expression	Pathways	Target
miR-221 ^9^	Increased	Cell cycle, proliferation, migration, apoptosis	p27Kip1, PTPμ, PUMA
miR-222 ^9^^,^^14^	Increased	Cell cycle, proliferation, migration, apoptosis	p27Kip1, PTPμ, PUMA
miR-210 ^10^	Increased	Proliferation, angiogenesis	FGFRL1, GPD1L
miR-15b ^11^	Decreased	Cell cycle, apoptosis, invasion, angiogenesis	cyclin D1, NRP, MMP-3
miR-21 ^12^^,^^14^	Increased	Angiogenesis, apoptosis, resistance to radio and chemotherapy	HIF-1a and VEGF, PDCD4, hMSH2
miR-125b ^13^	Increased	Growth and differentiation of CSCs, apoptosis	E2F2, p53, p38MAPK
miR 124-3p ^14^	Decreased	Cell cycle, angiogenesis	CDK6 kinase
miR-223 ^16^	Increased	Proliferation	IL-1β, MCP-1), IL-8 and IL-18

Eight studies were assessed ^9^^–^^16^ and 4 studies had a low risk of bias in the overall domain. ^9^^–^^11^^,^^16^ Meanwhile, three studies were concerned with qualitative measure. ^5^^,^^11^^,^^12^^,^^15^ One study has critical judgement. ^13^

Three studies had bias due to the selection of participants because of the variances of the participants’ characteristics. ^9^^,^^14^^,^^15^ One study had bias owing to confounding variables. ^14^ One studies had some bias due to missing data in some specific miRNA expression. ^9^ Four studies have some attrition bias because of many outcomes reported in the studies. ^11^^–^^14^ One study had a critical bias due to the heterogeneity of the participants. ^13^ Lastly, one study had a serious risk of bias due to the measurement mechanism of the outcomes. ^11^

A summary of the GRADE assessment for each study is presented in Table 3. Of the eight studies included in this systematic review, six were rated high and two were rated moderate. The true effect from each study was close to the estimate of the effect.

**Table 3.** Assessment of certainty of each evidence.

Certainty assessment								Effect	Quality of evidence (GRADE)
No	Journals	Study Desain	Risk of bias	Inconsistency	Indirectness	Imprecision	Other considerations	Relative (95% CI)	
1	Evgeny V. Stupak, 2023	Observational	Low	Not serious	Not serious	Not serious	None	HR 1.228 (1.073-1.407)	⊕⊕⊕⊕ **High**
2	Raffaela Barbano, 2014	Observational	Low	Not serious	Not serious	Not serious	None	HR 1.19 (1.01-1.41)	⊕⊕⊕⊕ **High**
3	Nian-sheng Lai, 2013	Observational	Low	Not serious	Not serious	Not serious	None	PFS p<0.001 (1.58-3.69)	⊕⊕⊕⊕ **High**
4	Xinxing Li, 2014	Observational	Low	Not serious	Not serious	Not serious	None	HR 2.43 (1.13-2.54)	⊕⊕⊕⊕ **High**
5	Debora Olioso, 2021	Observational	Low	Not serious	Not serious	Serious	None	PFS 8.5 (1-41)	⊕⊕⊕⊝ **Moderate**
6	Guan Sun, 2015	Observational	Low	Serious	Not serious	Not serious	None	Not Measured	⊕⊕⊕⊝ **Moderate**
7	Lin Wu, 2013	Observational	Low	Not serious	Not serious	Not serious	None	HR 3.17 (2.39-4.179)	⊕⊕⊕⊕ **High**
8	Rui Zhang, 2014	Observational	Low	Not serious	Not serious	Not serious	None	HR 2.81 (1.70-4.65)	⊕⊕⊕⊕ **High**
GRADE indicates Grading of Recomendations, Asessment, Development, and Evaluation; HR, Hazard Ratio; PFS, Progression-Free Survival									
1	High	We are very confident that the true effect lies close to that of the estimate of the effect.							
2	Moderate	We are moderately confident in the effect estimate: The true effect is likely to be close to the estimate of the effect, but there is a possibility that it is substantially different.							
3	Low	Our confidence in the effect estimate is limited: The true effect may be substantially different from the estimate of the effect.							
4	Very Low	We have very little confidence in the effect estimate: The true effect is likely to be substantially different from the estimate of effect.							

## 4. Discussion

Gliomas, the most prevalent and aggressive form of primary brain tumors in adults, continue to present a daunting challenge despite medical advancements. Currently, the treatment approaches for high-grade gliomas rely on surgical procedures, radiation therapy, and chemotherapy. Regrettably, these methods, whether used individually or in combination, struggle to effectively control the disease, resulting in an average life expectancy of just over 12-15 months post-diagnosis. ^2^ Consequently, the importance of timely diagnosis and prognostication remains unclear. The concept of molecular biomarkers has made substantial progress in the past decade, offering insights into glioma pathogenesis, aiding early tumor detection, risk assessment for recurrence, treatment strategy adjustments, and ultimately, improved prognosis. ^4^ Among which, circulating miRNAs have emerged as a prominent focus of research. Although not yet integrated into clinical practice, advancements in this domain suggest that circulating miRNAs may play a pivotal role in high-grade glioma diagnosis and prognosis, potentially replacing the specific elements of contemporary diagnostic procedures.

Our findings revealed that miR-210, miR15b, miR-21, miR-125b, miR 223 could be extracted from glioma tissues. miR-21, miR-221, miR-222, and miR-124-3p can be extracted from blood, indicating their potential as early diagnostic and prognostic tools for gliomas prior to surgery.

Different tissues were used as controls in glioma studies. Wu et al, ^12^ Li et al, ^13^ and Stupak et al, ^16^ used adjacent non tumor tissue from the same glioma patient. Lai et al, and Sun et al, used samples of normal brain tissue collected from the internal decompression surgery on patients with cerebral injury and cerebral hemorrhage. ^10^^,^^11^ Barbano et al, used RNAs from four healthy individuals consists different brain regions (parietal cortex, frontal cortex, occipital cortex, and striatum). ^15^ In using blood as a source of miRNA, there were also differences between different study. Zhang et al, used venous blood from other non-tumor patient. ^9^ While Olioso et al, used blood from the same patient which were taken repeatedly along the duration of radio- and chemotherapy. ^14^

As an overview, miRNA could serve as diagnostic, prognostic, and monitoring biomarker for high grade glioma. Zhang et al has demonstrated that miR-221 and miR-222 has good diagnostic value (AUC 0.83). ^9^ On the other hand, Olioso et al further demonstrated that miR-21, miR-222, and miR-123-3p can be used to predict response to therapy. High level of miR-21, -222 and -124-3p during post-operative follow-up was associated with tumor progression. miR-21 was the earliest biomarker to predict response of therapy at 17 week post op, with progression free survival ratio (high/low) 0.7 (CI 0.34 – 1.42). ^14^ As prognostic biomarker, Wu et al showed correlation between expression level of miR-21 and grading of glioma (p < 0.005). Kaplan-Meier survival curves suggested that survival time of glioma patients with high miR-21 was significantly shorter compared to those with low miR-21 expression (p < 0.001). Further analysis of each miRNA will be described below. ^12^

### 4.1 Role of miRNAs in glioma

*miR-221/miR-222*

The expression levels of miR-221 and miR-222 were higher in patients with gliomas than in the controls (P = 0.001). These miRNAs could potentially serve as biomarkers to distinguish glioma patients from controls, with an AUC of 0.83 (95% CI 0.74–0.93) for miR-221 and 0.88 (95% CI 0.79–0.98) for miR-222. The correlation between miR-221/222 expression and glioma prognosis may be partly explained by their role in promoting glioma cell proliferation through the downregulation of p27Kip1 expression. Additionally, miR-221/miR-222 are implicated in glioma cell migration by suppressing PTPμ expression and targeting apoptosis through direct binding to the 3’UTR mRNA region of the PUMA gene. ^9^^,^^14^

*miR-210*

The expression levels of miR-210 were significantly elevated in both high-grade (grade III–IV; 6.58 ± 1.42) and low-grade (grade I–II; 4.70 ± 0.93) gliomas compared to normal brain tissues (both p < 0.001). High-grade glioma tissues (III–IV) exhibited a significantly higher expression of miR-210 compared to low-grade tissues (I–II) (p < 0.001). miR-210 regulates glioma cell proliferation by targeting FGFRL 1, and under hypoxic conditions, it establishes a positive feedback loop that enhances HIF-1a stability by suppressing GPD1L, leading to the induction of genes associated with energy metabolism, angiogenesis, cell proliferation, and survival. ^10^

*miR-15b*

miR-15b expression in human glioma tissues was significantly lower than that in the adjacent normal brain tissues (p < 0.01). Moreover, the expression of miR-15b in both grade III and IV glioma tissues was significantly lower than that in grade I–II gliomas (p < 0.01). Overexpression of miR-15b inhibits proliferation by arresting cell cycle progression and inducing apoptosis by directly targeting cyclin D in gliomas. It was reported that miR-15b reduced glioma cell invasion and angiogenesis through NRP and MMP-3. ^11^

*miR-21*

miR-21 expression was significantly (P < 0.001) higher in glioma tissues (20.99 ± 13.04) than in the corresponding non-neoplastic brain tissues (0.73±0.05). miR-21 expression in high-grade (III-IV) gliomas was higher than that observed in low-grade (I-II) gliomas (P < 0.001).9 In the T98G glioma cell line, the level of expression of the PDCD4 gene showed an inverse relationship with the expression of miR-21, and its reduction led to the inhibition of apoptosis. Overexpression of miR-21 in DU145 cells leads to elevated levels of HIF-1a and VEGF, promoting tumor angiogenesis.15 Elevated serum miR-21 levels have been identified as an indicator of a poorer response to trastuzumab. Resistant response to radiotherapy and temozolamide has been demonstrated to be mediated by miR-21. ^12^

*miR-125b*

miR-125b revealed higher expression in glioma patients than controls (P < 0.001) and could potentially serve as a biomarker with an AUC of 0.83 (95% CI 0.74–0.93). miR-125b influences glioma stem cell proliferation by directly targeting E2F2. Additionally, it interacts with the 3′UTR of the apoptosis protein Bcl-2 modifying factor, inhibiting cell apoptosis through p53 and p38MAPK-independent pathways. ^13^

*miR-223*

The expression of miR-223 was significantly higher in grade IV glioma tissues than in adjacent normal tissues (p = 0.014), but no significant differences were observed between the different glioma grades. There is conflicting evidence regarding whether miR-223 acts as a tumor suppressor or oncogene in glioblastoma. Ding et al discovered that the overexpression of miR-223-3p hinder cell proliferation and migration in glioblastomas by controlling inflammation-associated cytokines. ^16^

*miR-124-3p*

miR-124-3p demonstrated lower expression in grade IV glioma patients compared to controls (P = 0.028), with no significant differences between different grades. Functioning as a tumor suppressor, miR-124 regulates the cell cycle at G0/G1 and inhibits CDK6 kinase, thereby suppressing angiogenesis and promoting neuronal differentiation. ^14^

### 4.2 Prognostic significance of MiRNA in high-grade glioma

Several miRNAs have shown promise as potential prognostic markers for high-grade gliomas, providing valuable insights into disease progression and patient outcome. High-grade gliomas, known for their aggressiveness, require accurate prognostic tools to guide their treatment. In this context, the Hazard Ratio (HR) is a critical statistical measure used to assess the prognostic significance of miRNAs. ^13^ HR represents the ratio of the risk of an event, such as disease progression or mortality, between two groups: one with high miRNA expression and the other with low expression. An HR greater than 1 indicates a higher risk of the event in the high-expression group, suggesting an unfavorable prognosis, while an HR less than 1 signifies a lower risk, implying a potentially better outcome. ^10^ By analyzing the HR associated with specific miRNAs, researchers can identify those that have a substantial impact on high-grade glioma prognosis. These miRNAs not only offer prognostic value but also hold the potential to guide personalized treatment strategies, ultimately improving patient care and outcomes in this challenging condition.

Eight miRNAs and their abilities to predict glioma prognosis were investigated in this study. Patients with high levels of miR-221, miR-222, miR-210, miR-21, miR-125b, and miR-223 and low levels of miR-15b and mir-124-3p expression had a significantly higher HR than those with low expression levels. HR represents the ratio of the risk of disease progression or mortality between groups with high and low miRNA expression.

The use of miRNAs as noninvasive biomarkers for the diagnosis and prognosis of high-grade gliomas offers several advantages. First, miRNAs can be easily detected in various bodily fluids, such as blood or cerebrospinal fluid, eliminating the need for invasive procedures such as brain biopsies. ^9^ This noninvasive approach not only reduces patient discomfort but also lowers the associated risks and costs. Secondly, miRNAs exhibit remarkable specificity, allowing the identification of glioma-specific miRNA signatures. This precision aids in accurate diagnosis and can potentially differentiate high-grade gliomas from other brain conditions, thereby enhancing the diagnostic certainty. Furthermore, miRNAs play critical roles in tumor development and progression, making them valuable indicators of disease aggressiveness and patient prognosis. ^14^ Their ability to reflect dynamic changes in tumor biology over time enables clinicians to tailor treatment strategies for individual patients. In summary, miRNAs serve as noninvasive, specific, and dynamic biomarkers that hold great promise for transforming the diagnosis and prognostication of high-grade glioma and improving patient care and outcomes.

Although assessing miRNA expression levels holds promise for predicting glioma prognosis, several challenges must be addressed before using miRNAs in clinical settings. First, cell-free miRNAs can be released from normal human tissues, potentially interfering with results. It is crucial to pinpoint the origin of tumor-specific miRNAs and develop a method capable of effectively distinguishing cancer populations from healthy individuals. Second, there is no standardized procedure for measuring miRNA levels, leading to conflicting findings. Furthermore, a single miRNA can be associated with various tumor types. ^2^ For instance, miR-21’s prognostic significance has been established in breast cancer, pancreatic ductal adenocarcinoma, and gastric cancer patients. ^4^ Therefore, identifying a set of miRNAs specific to glioma holds great promise and could significantly enhance prognostic accuracy.

### 4.3 Strengths and limitations

The strength of this review article lies in its comprehensive assessment of both the diagnostic and prognostic approaches for miRNAs in the context of various medical conditions. By covering a wide spectrum of applications, this study offers a holistic view of the potential use of miRNAs in medicine. This is validated by country variability, study centers, and various patient characteristics, which suggest broad generalizability. Additionally, the studies included in this review were meticulously evaluated using standardized parameters to ensure the reliability and consistency of the findings. This finding was coupled with the specific types, targets, and mechanisms of each potential miRNA included in the highlighted findings. This rigorous approach enhanced the credibility of the conclusions and recommendations of this study.

This study had some limitations. One notable limitation was the scarcity of studies that met the eligibility criteria for inclusion. The paucity of relevant research may limit the breadth of the review and ability to draw definitive conclusions regarding certain aspects of the diagnostic and prognostic utility of miRNAs. This scarcity may also introduce potential bias in the interpretation of available data. A quantitative review with a larger sample size is required to verify the statistical significance of the expression of each miRNA. Despite these limitations, this review provides valuable insights into the current state of knowledge on the diagnostic and prognostic potential of miRNAs.

## Ethics and consent

Ethical approval and consent were not required.

## Data Availability

No data is associated with this article. Figshare: PRISMA checklist for ‘Diagnostic and prognostic values of miRNAs in high-grade gliomas: A systematic review’. https://doi.org/10.6084/m9.figshare.26140279 Data are available under the terms of the Creative Commons Zero “No rights reserved” data waiver (CC0 1.0 Public domain dedication).
