# Supplementary material for: Diagnostic and Prognostic Values of miRNAs in High-Grade Gliomas: A Systematic Review
Source: F1000Res. 2025 Jan 15;13:796. Originally published 2024 Jul 15. [Version 2] doi: 10.12688/f1000research.151350.2 (PMC11826073; doi:10.12688/f1000research.151350.2)
Supplement: Peer review history

# Reviewer report: Reviewer response for version 2

**DOI:** 10.5256/f1000research.176321.r364631

**Published:** 2025-02-13

**Authors:** Abel Po-Hao Huang

**Affiliations:** 1 National Taiwan University Hospital, Taipei, Taiwan

This article indicates that specific miRNAs, such as miR-221, miR-222, and miR-21, are associated with poor prognosis and could serve as valuable indicators of disease progression.

 The article emphasizes the need for further validation through extensive clinical trials to fully realize the clinical utility of these biomarkers, which could ultimately improve patient outcomes in the context of high-grade gliomas.

 This is a well-written review which had meet stringent requirements of high scientific quality and significance.

Are the rationale for, and objectives of, the Systematic Review clearly stated?

Yes

Is the statistical analysis and its interpretation appropriate?

Yes

If this is a Living Systematic Review, is the ‘living’ method appropriate and is the search schedule clearly defined and justified? (‘Living Systematic Review’ or a variation of this term should be included in the title.)

Not applicable

Are sufficient details of the methods and analysis provided to allow replication by others?

Yes

Are the conclusions drawn adequately supported by the results presented in the review?

Yes

Reviewer Expertise:

ICH, Minimally invasive neurosurgery (MIN) , Neuroprotection & Neural repair

I confirm that I have read this submission and believe that I have an appropriate level of expertise to confirm that it is of an acceptable scientific standard.

# Reviewer report: Reviewer response for version 2

**DOI:** 10.5256/f1000research.176321.r359383

**Published:** 2025-01-23

**Authors:** Dessika Rahmawati

**Affiliations:** 1 Universitas Brawijaya/Dr. Saiful Anwar General Hospital, Malang, Indonesia

Dear Journal Editor 

 I accept the review of this journal and I agree to index this journal without more revision. 

 Thankyou

Are the rationale for, and objectives of, the Systematic Review clearly stated?

Yes

Is the statistical analysis and its interpretation appropriate?

I cannot comment. A qualified statistician is required.

If this is a Living Systematic Review, is the ‘living’ method appropriate and is the search schedule clearly defined and justified? (‘Living Systematic Review’ or a variation of this term should be included in the title.)

Yes

Are sufficient details of the methods and analysis provided to allow replication by others?

Partly

Are the conclusions drawn adequately supported by the results presented in the review?

Yes

Reviewer Expertise:

In essence, this review is crucial because it not only advances our understanding of gliomas at a molecular level but also has the potential to translate these findings into tangible benefits for patients. It represents a bridge between cutting-edge research and clinical application, with the ultimate goal of improving outcomes for individuals affected by this challenging disease.

I confirm that I have read this submission and believe that I have an appropriate level of expertise to confirm that it is of an acceptable scientific standard.

# Reviewer report: Reviewer response for version 1

**DOI:** 10.5256/f1000research.165992.r340043

**Published:** 2024-12-12

**Authors:** Dessika Rahmawati

**Affiliations:** 1 Universitas Brawijaya/Dr. Saiful Anwar General Hospital, Malang, Indonesia

Feedback:

- Ensure consistent use of terminology (e.g., "miRNA" vs. "mRNA" in the context of gliomas).
- **Citations:** Verify all citations for accuracy and completeness. For instance, ensure the references like "1" and "2" directly correspond to listed sources.
- **Methodology:** Your methods section is detailed, but consider adding a flowchart or diagram to visually represent the selection and inclusion process.
- **Results and Discussion:** If possible, include a brief overview of key findings from the included studies. Highlight any significant miRNA biomarkers identified and their clinical implications.

Overall, this article provides valuable insights into the role of miRNAs in glioma diagnosis and prognosis. It would be beneficial to ensure that all sections are seamlessly integrated, with a clear connection between the background, methods, results, and conclusions.

Are the rationale for, and objectives of, the Systematic Review clearly stated?

Yes

Is the statistical analysis and its interpretation appropriate?

I cannot comment. A qualified statistician is required.

If this is a Living Systematic Review, is the ‘living’ method appropriate and is the search schedule clearly defined and justified? (‘Living Systematic Review’ or a variation of this term should be included in the title.)

Yes

Are sufficient details of the methods and analysis provided to allow replication by others?

Partly

Are the conclusions drawn adequately supported by the results presented in the review?

Yes

Reviewer Expertise:

In essence, this review is crucial because it not only advances our understanding of gliomas at a molecular level but also has the potential to translate these findings into tangible benefits for patients. It represents a bridge between cutting-edge research and clinical application, with the ultimate goal of improving outcomes for individuals affected by this challenging disease.

I confirm that I have read this submission and believe that I have an appropriate level of expertise to confirm that it is of an acceptable scientific standard, however I have significant reservations, as outlined above.

# Response

**Published:** 2024-12-24

**Authors:** Renindra Ananda Aman

**Affiliations:** Neurosurgery, Rumah Sakit Dr Cipto Mangunkusumo, Central Jakarta, Jakarta, Indonesia

1.⁠ ⁠We have make sure that terminology miRNA is the one that we use in our study, not mRNA

 2.⁠ ⁠At method section, we add one sentence that explain the flow chart will be displayed at result section.

 3.⁠ ⁠From result until discussion section, we have adjust and verify all citations used in our study

 4.⁠ ⁠At discussion section, one paragraph has been added explaining brief overview and highlight of significant miRNA
